# Hemoglobin and Neutrophil Count as Prognostic Factors in Cholangiocarcinoma Patients in 2nd Line Treatment Setting: Results from a Small Monocentric Retrospective Study

**DOI:** 10.3390/curroncol30010079

**Published:** 2023-01-11

**Authors:** Carolina Liguori, Cecilia Copparoni, Cristiano Felicetti, Federica Pecci, Alessio Lupi, Giada Pinterpe, Rossana Berardi, Riccardo Giampieri

**Affiliations:** 1 Clinica Oncologica, Dipartimento Scienze Cliniche e Molecolari, Università Politecnica delle Marche, 60121 Ancona, Italy; 2 Clinica Oncologica, Azienda Ospedaliero Universitaria delle Marche, 60126 Ancona, Italy

**Keywords:** cholangiocarcinoma, hemoglobin, neutrophil count, prognostic, 2nd line chemotherapy

## Abstract

Background: Unresectable cholangiocarcinoma prognosis can be extremely variable due to different symptoms and sites of disease involvement at diagnosis and unpredictable chemotherapy response rates. Most patients will usually receive 1st line palliative chemotherapy with platinum compounds and Gemcitabine or Gemcitabine alone. Only a few patients maintain adequate performance status after first-line treatment failure: second-line treatment with FOLFOX or FOLFIRI chemotherapy has been used in this setting with modest overall survival improvement. There is a lack of data concerning whether laboratory findings might help clinicians in identifying those patients with the highest likelihood of benefiting from 2nd line treatment. The aim of this analysis is to assess the prognostic role of a series of easily available laboratory tests in patients with bile duct cancer who received 2nd line chemotherapy. Patients and Methods: Patients with unresectable bile duct cancer treated in 2nd-line setting with platinum-based chemotherapy doublet or FOLFIRI were enrolled. The primary objective of the analysis was to assess overall survival (OS) differences among patients based on the results of lab tests. Serum hemoglobin, neutrophil, lymphocyte, monocyte, platelet absolute count, creatinine, total bilirubin, albumin, LDH, circulating CEA and CA19.9 values were collected at the start of 2nd line treatment. Cut-off values for all lab tests were set by ROC curve analysis. Survival was calculated by the Kaplan–Meier method and differences in survival among stratification factors were assessed by Log-rank test. Cox-proportional-hazard regression was used for multivariate analysis. Level of statistical significance *p* was set at 0.05 for all tests. Correction for false discovery error rate was performed by Holm’s stepdown procedure. Results: A total of 46 patients were eligible. Median overall survival of the entire cohort was 8.98 months (95%CI: 6.68–13.93) while mean OS was 17.10 months (standard error: 3.16). Using 6.2 months OS landmark as classification variable for ROC curve analysis, only serum hemoglobin (cut-off: >10 g/dL), albumin (cut-off: >3.5 mg/dL), CA19.9 (cut-off: ≤668 UI/mL), monocyte (cut-off: ≤510/mmc) and neutrophil count (cut-off: ≤5140/mmc) were significantly associated with the chosen end-point. Multivariate analysis confirmed an independent statistically significant impact on overall survival only for hemoglobin (Exp(b): 0.12, *p* = 0.0023) and neutrophil count (Exp(b): 0.30, *p* = 0.0039). Based on these results, using both hemoglobin and neutrophil count, three prognostic groups were defined: patients with both favorable factors had 12.63 months median OS vs. 6.75 months of patients with only one favorable factor vs. 1.31 months of those with neither. The difference between these three groups of patients was statistically significant (*p* < 0.0001). Discussion: Second-line palliative chemotherapy can be a potentially useful option for a few patients with unresectable/metastatic bile duct cancer. Even though assessment of patients’ prognosis might be difficult due to the complex behavior of this disease, a series of easily available laboratory tests might be used for these means: serum hemoglobin and neutrophil count we0re able to define subsets of patients with entirely different prognoses. It is hoped that this score will be prospectively validated in a larger group of patients in order to improve treatment decisions in patients with unresectable bile duct cancer candidate to receive palliative 2nd line chemotherapy.

## 1. Introduction

Biliary tract cancers (BTC) are a heterogeneous family of rare neoplasms that includes gallbladder cancer, ampullary cancer, and intrahepatic, hilar, and extrahepatic cholangiocarcinoma. Although surgery provides the only possibility of cure, most cases are diagnosed in advanced stages of disease involvement [1]. Cisplatin/gemcitabine combination has represented the standard of care as first-line treatment [2], yielding modest survival advantage at best. Recently, Durvalumab, an anti-PD1 checkpoint inhibitor, was used in combination with first-line chemotherapy with Cisplatin/gemcitabine and has improved these results, leading to 12.9 months of median overall survival (OS) for these patients. Despite that, the majority of patients will ultimately progress during first-line treatment; some of them still maintain adequate performance status after first-line treatment failure, and 30–35% are able to receive also second-line treatment [3].

Treatment options after first-line progression are less defined: ABC-06 trial, an open-label, randomized, multicenter trial, that compared fluorouracil and oxaliplatin (mFOLFOX: Oxaliplatin 85 mg/m^2^, L-folinic acid 175 mg/m^2^ or folinic acid 350 mg/m^2^, 5-Fluorouracil bolus 400 mg/m^2^ and 5-Fluorouracil continuous infusion 2400 mg/m^2^ every two weeks) plus active symptom control (ASC) with ASC alone in second-line setting has proven that mFOLFOX chemotherapy might improve patients’ survival also in a second-line setting; OS was significantly longer in the ASC plus mFOLFOX group compared to ASC alone. Median OS were, respectively, 6.2 months in the ASC plus mFOLFOX group versus 5.3 months in the ASC alone group [4]. These results provided evidence of survival benefit when using 2nd line treatment with mFOLFOX over best supportive care and rapidly became the new standard of care.

A recent multicenter retrospective analysis by Mizrahi et al. [5] assessed the role of fluorouracil and irinotecan (FOLFIRI: Irinotecan 180 mg/m^2^, L-folinic acid 200 mg/m^2^ or folinic acid 400 mg/m^2^, 5-Fluorouracil bolus 400 mg/m^2^ and 5-Fluorouracil continuous infusion 2400 mg/m^2^ every two weeks) in patient with advanced BTC as 1st, 2nd, 3rd or 4th–nth line therapy. In particular, median PFS and OS of patients treated with FOLFIRI in second-line were, respectively, 2.4 (95%CI: 1.8–3.7) and 7.7 (95%CI: 4.9–10.5) months. These survival outcomes were similar to historical controls of other retrospectively examined second-line cytotoxic therapy options.

As it can be seen, despite growing interest in discovering new treatment options for patients with metastatic/unresectable cholangiocarcinoma, most of them are based on a standard mechanism of action of “old” antineoplastic chemotherapeutic drugs: in particular, 5-Fluorouracil main mechanism of action is based on thymidylate synthase inhibition, thus disrupting tumor cell DNA synthesis; Oxaliplatin, on the other hand, behaves like a alkylating antineoplastic agent, thus causing damage by covalent DNA binding and causing DNA double-strand breaks (particularly in tumors that might have impaired homologous recombination system defects such as in BRCA mutated tumors); finally, Irinotecan (CPT-11) is converted into its active form (SN-38) that binds to topoisomerase-I enzyme, thus preventing relegation of DNA strand and causing lethal double-strand breaks. Based on these mechanisms of action, damage to normal healthy cells can be expected and thus, factors able to identify which patients can benefit from second-line treatment or would not be able to tolerate said treatment is a relevant clinical issue.

During the last few years, several factors have shown to be correlated with better survival of patients treated with second-line therapy, such as Eastern Cooperative Oncology Group (ECOG) performance status (PS) of 0, CA19.9 serum level ≤152 Um^−1^, previous surgery on primary tumors and PFS after first-line chemotherapy ≥ 6 months [6], absence of peritoneal carcinomatosis and toxicity as reason for first-line therapy discontinuation rather than disease progression [7]. However, the capability of these models to accurately predict prognosis is limited and their usefulness in predicting second-line treatment outcome remains unclear.

Inflammation has been documented to play decisive roles in cancer development and progression in a variety of human cancer [8], including cholangiocarcinogenesis and progression of BTC [9,10], raising the interest around host inflammatory and immune status as predictors of outcome. Routine baseline blood parameters are able to represent patient systemic inflammation in a reliable and easily accessible way. Cancer cells are able to produce cytokines such as granulocyte colony-stimulating factor (G-CSF) and IL-6, thus promoting neutrophils proliferation. On the other hand, neutrophils are able to produce growth factors enhancing cancer cell tumorigenicity and promote circulating tumor cells endothelium adhesion and extravasation, thus promoting tumor metastases [11,12]. Lymphocytes on the other hand are among the most important factors involved in anti-cancer immunity; it has been previously reported that lymphopaenia is related to shorter survival and lower response to treatment in several malignancies due to immune system failure against tumor growth [13]. Finally, upper limit normal LDH, low albumin levels and high neutrophil to lymphocyte ratio (NLR) have already been associated with a worse outcome in different types of cancer patients [14]. Based on these results many prognostic scores that consider baseline serum levels of these blood tests have already been developed in different cancer settings, as they provide a useful tool for clinicians’ everyday decision-making [15,16].

In this retrospective monocentric study, we assessed the prognostic value of lab test results in a small group of BTC patients treated with second-line chemotherapy with the aim of developing a prognostic model aiding patients’ stratification as to identify those patients who have better prognosis and would be the best candidates to receive further treatment options after first-line palliative treatment progression.

## 2. Materials and Methods

### 2.1. Patients

We consecutively enrolled in this retrospective analysis patients treated with 2nd line palliative chemotherapy for an unresectable locally advanced/metastatic bile duct cancer, treated at our Institution, from 2010–2022.

Patients eligible for analysis should have received 1st line palliative chemotherapy with chemotherapy doublet comprising platinum compound (cisplatin/oxaliplatin) and/or either gemcitabine or fluoropyrimidine analogs.

No upper/lower age boundaries were set and no restriction on the chemotherapy regimen was set as well. We did not include in this analysis patients who received in 2nd line setting targeted therapies or immunotherapy (such as Pemigatinib for FGFR2 alteration carriers, Ivosidenib for IDH1 mutated patients and Dabrafenib+Trametinib combo for BRAF V600E mutated patients) as the clinical behavior in these instances could be different compared to those patients whose tumors do not harbor these mutations.

### 2.2. Methods

For all patients included in the analysis, baseline (date of the first cycle of chemotherapy ±7 days) serum levels of the following lab tests were collected: hemoglobin, neutrophil, lymphocyte, monocyte, platelet absolute count, creatinine, bilirubin, albumin, LDH, circulating CEA and CA19.9 values. Normality of distribution was assessed by Shapiro–Wilk test: this test was chosen instead of Kolmogorov–Smirnov or D’Agostino test as it has better power to assess normality of distribution particularly whenever the sample size is rather small such as in our study.

All lab tests were performed as follows: complete blood count with differential was performed by fluorescence flow cytometry on a Sysmex XN-9100 automated hematology system (Dasit Group, Milan, Italy). Serum creatinine (IFCC-standardized enzymatic method), total (Jendrassik-Grof) and direct (diazotisation) bilirubin, AST/ALT (IFCC-calibrated UV method with pyridoxal-5-Phosphate), and albumin (bromocresol red) were measured by photo absorptiometry on a Siemens Dimension Vista 1500 automated clinical chemistry analyzer (Siemens Healthineers, Erlangen, Germany). CEA and CA19.9 were measured on a Siemens Dimension Vista 1500 analyzer using automated sandwich chemiluminescent immunoassays based on the LOCI^®^ technology.

We expected that lab tests results might not follow normal distribution: because of that median value of distribution would not be considered adequate as an optimal cut-off point. Instead, we opted for ROC curve analysis as a means to identify optimal cut-off values. For each lab test, we calculated optimal cut-off by using as classification variable the fact of having achieved at least 6.2 months OS: this classification variable was used as it corresponds to the median OS of the mFOLFOX+ASC treatment arm of the ABC-06 trial; we sought to identify those patients who would have survival outcomes on par or superior to those patients. The cut-off was based on Youden index J.

Due to the rarity of data concerning risk stratification in this subset of patients, adequate sample-size estimation is not possible. However, if we hypothesis that, at 6.2-overall survival timepoint, patients with positive prognostic features will have a 66% overall survival rate, compared to only 20% of those patients with negative prognostic features, with alfa-error probability set at 0.05 and beta-error probability set at 0.20, if patients with negative and positive prognostic features are evenly distributed, 40 patients (20 in each prognostic group) are required to test this hypothesis.

We defined overall survival as the time from beginning of 2nd line treatment until death for whichever cause or lost at follow-up visit. Survival was calculated by Kaplan–Meier method and differences in survival among stratification factors were assessed by a Log-rank test.

As changes in different lab-tests might be correlated (as in the corresponding increase/decrease in neutrophil and monocyte count per example), we used Cox-proportional-hazard regression multivariate analysis as to sort out their independent impact on OS. Only variables that were associated at univariate analysis with a statistically significant impact on OS were included in the model.

Level of statistical significance *p* was set at 0.05 for all tests. Appropriate correction for false discovery error rate was performed by Holm’s stepdown procedure.

All calculation were performed by using R software (version 4.1.2, copyright The R Foundation for Statistical Computing, packages MatchIt, Survminer, Survival, ROCit) and MedCalc^®^ Statistical Software version 19.7.2 (MedCalc Software Ltd., Ostend, Belgium; https://www.medcalc.org; 2021).

## 3. Results

### 3.1. Patients’ Characteristics and Laboratory Tests Results

**Figure 1:**
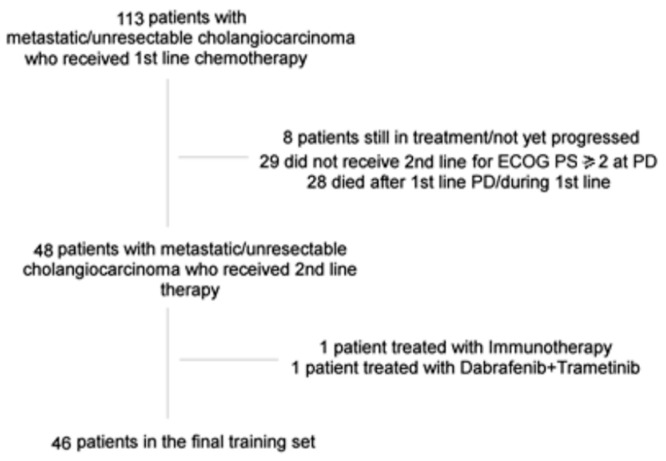
Patients’ selection flow diagram.

Forty-six patients were enrolled for analysis. Main patients’ characteristics are summarized in Table 1 while flow-diagram describing patients’ selection can be found in Figure 1.

Median overall survival of the entire cohort was 8.98 months (95%CI: 6.68–13.93) while mean OS was 17.10 months (standard error: 3.16). At the time of the present analysis, after a median follow-up time of 16.45 months, 26/46 (56%) patients had already died.

Median hemoglobin level was 11.45 g/dL (range: 8.7–15.7 g/dL). Hemoglobin values were normally deviated (*p* = 0.71).

Median neutrophil count was 4495/mmc (range: 1820–21,270/mmc). Neutrophil count was not normally deviated (*p* < 0.0001).

Median monocyte count was 525/mmc (range: 260–1220/mmc). Monocyte count was not normally deviated (*p* = 0.0011).

Median lymphocyte count was 1210/mmc (range: 470–2880/mmc). Lymphocyte count was not normally deviated (*p* = 0.0138).

Median platelet count was 194,500/mmc (range: 96,000–571,000/mmc). Platelet count was not normally deviated (*p* = 0.0017).

Median serum creatinine level was 0.83 mg/dL (range: 0.41–1.36 mg/dL). Serum creatinine was normally deviated (*p* = 0.15).

Median total bilirubin level was 0.45 mg/dL (range: 0.2–1.5 mg/dL). Bilirubin was not normally deviated (*p* < 0.0001).

Median serum albumin level was 3.6 mg/dL (range: 2.1–4.5 mg/dL). Serum albumin was not normally deviated (*p* = 0.0171).

Median LDH was 219 U/L (range: 96–701 U/L). LDH was not normally deviated (*p* < 0.0001).

Median CEA was 3.05 ng/mL (range: 0.3–246 ng/mL). CEA was not normally deviated (*p* < 0.0001).

Median CA19.9 was 206 U/mL (range: 2–100,000 U/mL). CA19.9 was not normally deviated (*p* < 0.0001).

A summary of laboratory tests distribution and deviations can be found in Table 2.

### 3.2. ROC Curve Analysis Results

**Figure 2:**
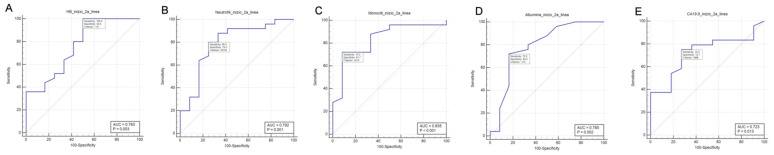
Result from ROC curve analysis. (**A**) Hemoglobin. (**B**) Neutrophils. (**C**) Monocytes. (**D**) Albumin. (**E**) CA19.9.

As already stated, ROC curve analysis was performed by using 6.2 months OS landmark as a classification variable. As a result of that:

Hemoglobin values were associated with different OS (*p* = 0.0031, AUC: 0.731) (Figure 2A). The best cut-off by Youden J-index was >10 g/dL. This cut-off was associated with 100% sensitivity and 50% specificity.

Neutrophil count was associated with different OS (*p* = 0.001, AUC: 0.792) (Figure 2B). The best cut-off by Youden J-index was ≤5140/mmc. This cut-off was associated with 80% sensitivity and 75% specificity.

Monocyte count was associated with different OS (*p* < 0.001, AUC: 0.835) (Figure 2C). The best cut-off by Youden J-index was ≤510/mmc. This cut-off was associated with 72% sensitivity and 91.7% specificity.

Lymphocyte count was not associated with differences in OS (*p* = 0.42, AUC: 0.580).

Platelet count was not associated differences in OS (*p* = 0.80, AUC: 0.525).

Serum creatinine was not associated with differences in OS (*p* = 0.13, AUC: 0.667).

Bilirubin was not associated with differences in OS (*p* = 0.17, AUC: 0.625).

Serum albumin was associated with differences in OS (*p* = 0.002, AUC: 0.785) (Figure 2D). The best cut-off by Youden J-index was >3.5 mg/dL. This cut-off was associated with 72% sensitivity and 83% specificity.

LDH levels were not associated with differences in OS (*p* = 0.69, AUC: 0.547).

CEA levels were not associated with 6.2 months OS (*p* = 0.06, AUC: 0.697).

Finally, CA19.9 levels were associated with 6.2 months OS (*p* = 0.013, AUC: 0.723) (Figure 2E). The best cut-off by Youden J-index was ≤688 U/mL. This cut-off was associated with 75% sensitivity and 72.7% specificity.

A summary of the results of ROC curve analysis can be found in Table 3.

### 3.3. Results of Univariate Analysis for OS

**Figure 3:**
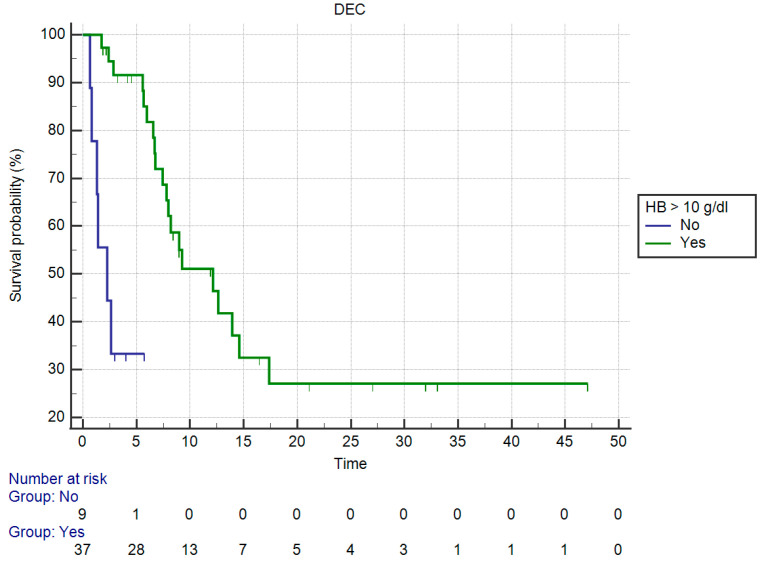
Kaplan–Meier curve for OS (Hemoglobin).

Overall survival (OS) in patients having HB > 10 g/dL vs. those having HB values ≤ 10 g/dL was significantly different (mOS, respectively, 12.31 vs. 2.26 months, HR: 0.014, 95%CI: 0.002–0.09, *p* < 0.0001 against corrected *p* = 0.01 by Holm stepdown procedure) (Figure 3).

**Figure 4:**
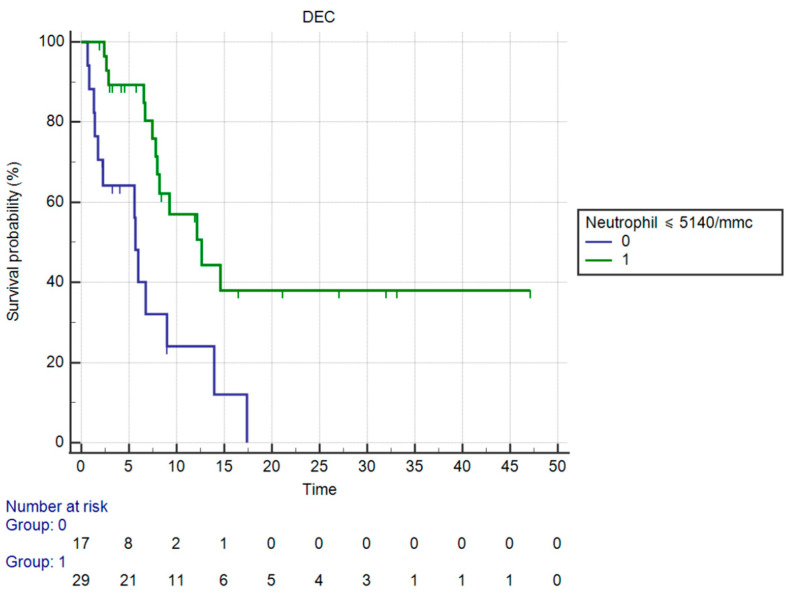
Kaplan–Meier curve for OS (Neutrophil count).

OS in patients having ≤5140 neutrophil count vs. those having >5140 neutrophils was significantly different (mOS, respectively, 12.62 vs. 5.67 months, HR: 0.23, 95%CI: 0.09–0.58, *p* < 0.0001 against corrected *p* = 0.0125 by Holm stepdown procedure) (Figure 4).

**Figure 5:**
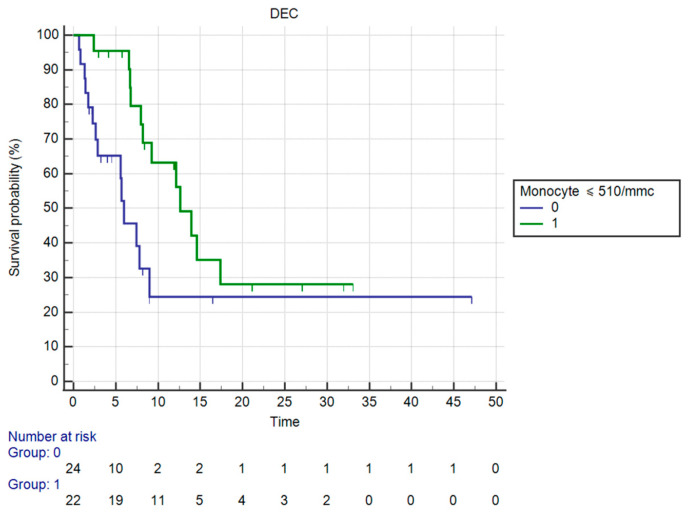
Kaplan–Meier curve for OS (Monocytes).

OS in patients having ≤510 monocyte count vs. those having >510 monocytes was significantly different (mOS, respectively, 12.62 vs. 5.97 months, HR: 0.36, 95%CI: 0.15–0.84, *p* = 0.0189 against corrected *p* = 0.025 by Holm stepdown procedure) (Figure 5).

**Figure 6:**
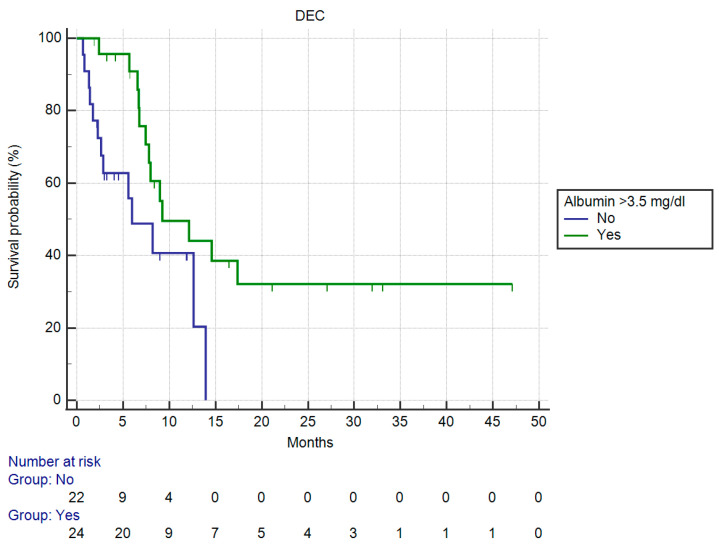
Kaplan–Meier curve for OS (Monocytes).

OS in patients having >3.5 mg/dL serum albumin vs. those having ≤3.5 mg/dL serum albumin was significantly different (mOS, respectively, 9.24 vs. 5.97 months, HR: 0.38, 95%CI: 0.16–0.91, *p* = 0.0302 against uncorrected *p* = 0.05 by Holm stepdown procedure) (Figure 6).

**Figure 7:**
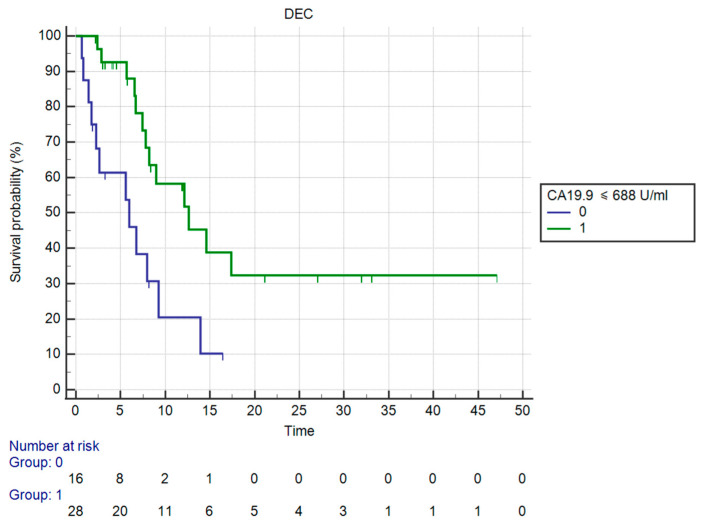
Kaplan–Meier curve for OS (Monocytes).

OS in patients having ≤688 U/mL CA19.9 levels vs. those having >688 U/mL CA19.9 was significantly different (mOS, respectively, 12.62 vs. 5.97 months, HR: 0.27, 95%CI: 0.10–0.68, *p* = 0.0057 against corrected *p* = 0.0166 by Holm stepdown procedure) (Figure 7).

### 3.4. Results of Multivariate Analysis

Results of the multivariate analysis can be seen in Table 4.

Overall model fitness was statistically significant (*p* < 0.0001) by including only serum hemoglobin levels greater than 10 g/dL (Exp(b): 0.12, *p* = 0.0023) and absolute neutrophil count lower than or equal to 5140/mmc (Exp(b): 0.30, *p* = 0.0039). On the other hand, serum albumin, monocyte count, CA19.9 levels lost their independent role as prognostic factors.

Based on these results we defined three “subgroups” of patients, as in those having both serum hemoglobin greater than 10 g/dL and absolute neutrophil count lower than or equal to 5140/mmc, those with only one favorable factor and those with neither.

**Figure 8:**
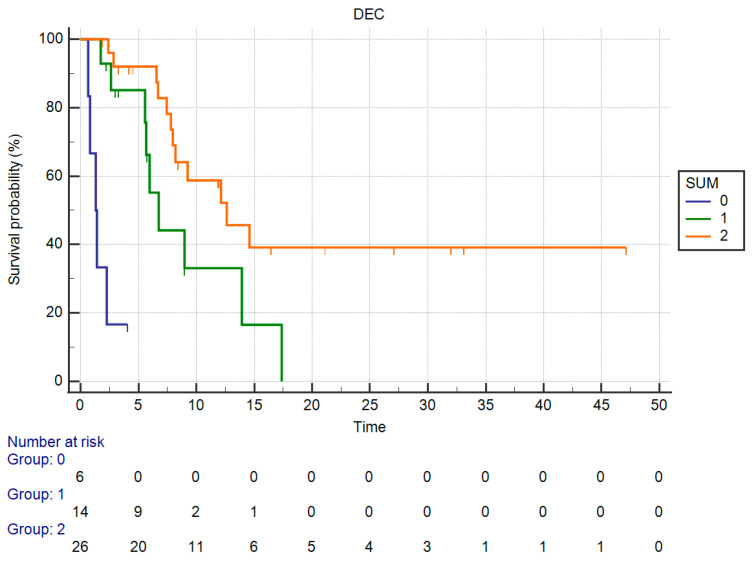
Kaplan–Meier curve for OS (hemoglobin + neutrophiles count).

OS in these three groups of patients were significantly different: in particular, median OS in those having both favorable factors was 12.62 months vs. 6.75 months of the group having only one factor vs. 1.31 months in the group of patients with neither (*p* < 0.0001) (Figure 8).

## 4. Discussion

Second-line palliative chemotherapy represents a potentially useful option for patients with advanced biliary tract cancer who still have good performance status after first-line treatment failure. We analyzed overall survival of patients treated with second-line chemotherapy in our Institution, evaluating whether different results of baseline lab tests might imply differences in patients’ prognosis.

The addition of FOLFOX to ASC improves median overall survival in patients with advanced biliary tract cancer after progression on cisplatin and gemcitabine, with a clinically meaningful increase in 6 months and 12 months overall survival rates [4]. The survival times reported in our study are marginally better but comparable to the mOS of 6.2 months (95% CI 5.4–7.6) of patients treated in 2nd line therapy with FOLFOX+ASC in the ABC-06 trial [4], since median overall survival of our entire cohort was 8.98 months. We believe that selection bias, due to the retrospective nature of our analysis, might be accounted for this difference.

Regarding prognostic factors, multivariate analysis showed that serum hemoglobin levels greater than 10 g/dL and absolute neutrophil count lower than or equal to 5140/mmc have a favorable prognostic value.

Cancer-related anemia is a common comorbidity in cancer patients and pathogenesis is related to a complex interaction between tumor cells and the immune system mediated by cytokines. Overexpression of several pro-inflammatory cytokines, such as IFN-γ, TNF-α, IL-1, and IL-6, results in an increased number of destroyed erythrocytes, suppression of erythropoiesis, due to iron restriction and impaired proliferation of erythroid progenitor cells [17,18]. In addition, chemotherapy itself contributes to decreased circulating erythrocytes due to its cytotoxic effect, with an incidence between 30% and 90% depending on the tumor type and hemoglobin cut-off considered [19].

The low level of hemoglobin is correlated with worsening of quality of life and with poor response to treatments and decreased survival, especially in the late-stage of disease [20]. It follows that chemotherapy dose reductions, iron supplementation and even treatment with recombinant human erythropoietin are often necessary in order to relieve symptoms and maintain treatment schedule [19,21].

Leukocytosis is relatively common in patients with solid tumors [22] and neutrophils represent the main population of leukocytes in the blood. Many patients with neutrophilia presented a more advanced stage of disease than the patients who did not have high blood neutrophil count [23], but the biological mechanisms leading to cancer-related neutrophilia are still uncertain. Therefore, this suggests that neutrophilia may be a surrogate for advanced disease burden not always described by stage classification.

Neutrophils are major inflammatory cells in the tumor microenvironment in several types of cancer, including cholangiocarcinoma [24]. Circulating neutrophils are recruited to the tumor microenvironment; moreover, neutrophils within the tumor tissue differentiate into tumor-associated neutrophils (TANs). These immune cells mainly present a protumorigenic (N2) phenotype and are mostly activated by TGF-b [25], released by tumor cells, and facilitate tumor-promoting effects. TANs provide a favorable environment for cancer development, angiogenesis and metastatic spread by secreting cytokines and chemokines, such as interleukin-2 (IL-2), interleukin-6 (IL-6), interleukin-10 (IL-10), tumor necrosis factor α (TNF-α) and vascular endothelial growth factor (VEGF) [26,27]. Moreover, TANs mediate immune editing and modulation of other tumor-infiltrating immune cells, affecting the possibility of the immune system to influence tumor growth. In particular, TANs can activate the immune escape mechanism inhibiting the anti-tumor properties of CD8+ T lymphocytes [28]. A recent study in pancreatic cancer patients explored the correlations between neutrophilia and TANs and has shown that high circulating neutrophil counts may be associated with intratumoral neutrophils frequency [29], but the phenotypic association between them remains an important open question.

High levels of circulating neutrophils were also shown to correlate with an increased risk for cancer-related thrombosis [30]: neutrophils release of pro-inflammatory cytokines and expression of molecules such as P-selectin and Tissue Factor on their membrane and on neutrophil-derived microvescicles plays a key role in thrombus generation [31]. Neutrophil can also expel their nuclear contents to form networks of decondensed chromatin known as Neutrophil Extracellular Traps (NETs) that bind pathogenic microbes but can also capture coagulation factors promoting thrombus formation [31,32].

Histologic studies have shown that high levels of intratumoral neutrophils are correlated with tumor recurrence, metastases and decreased survival and neutrophilia is an independent prognostic factor for CCA patients [33,34].

According to literature [35,36], our data suggest that also circulating neutrophils are an independent prognostic factor that can be easily studied in CCA patients.

In our study, the combination of both hemoglobin and neutrophil count enables to define three prognostic groups, with clearly separated OS: median OS for patients who did have both favorable factors was 12.62 months, whereas mOS was 6.75 months in the group of patients with only one favorable factor and 1.31 months of the group with neither. This fact suggests that assessment of these lab tests might help in predicting patients’ prognosis in a clinical setting where other features that might help treatment decisions are lacking.

We acknowledge the limitations of our study: the small number of patients requires further analyses in a larger cohort of patients in order to confirm our results. The monocentric nature of this study also contributes to this fact, as it requires external validation by repeating these analyses in other different laboratories. In addition to that, the retrospective nature of the study might have determined selection biases that might act as confounding factor and that can only be accounted for when these assessments are validated prospectively.

## 5. Conclusions

We still lack reliable prognostic factors other than ECOG performance status in order to estimate the life expectancy of patients with unresectable/metastatic cholangiocarcinoma who are candidates to receive 2nd line palliative treatment. The results of our retrospective analysis suggest that hemoglobin and neutrophil count might identify those patients who have worse survival outcomes. Should our results be confirmed in larger, prospectively conducted multicentric analyses, hemoglobin and neutrophil count might represent an easy-to-access and affordable tool to identify those patients who have the highest likelihood to benefit from 2nd-line chemotherapy.

## Figures and Tables

**Table 1:** Summary of patients’ characteristics.

Characteristics, *N* (%)	All Patients *N* = 46 (%)
Sex	
Male	28 (61)
Female	18 (39)
Age	
<65 years	15 (33)
>65 years	31 (67)
ECOG PS at the beginning of 2nd line treatment	
0–1	43 (93)
>2	3 (7)
Tumor location	
Intrahepatic	23 (50)
Perihilar cholangiocarcinoma	11 (24)
Extrahepatic	8 (17)
Gall bladder	4 (9)
Synchronous metastases	
Yes	23 (50)
No	23 (50)
Primary tumor resection	
Yes; received adjuvant treatment	16; 10 (34)
No	30 (66)
1st line chemotherapy	
CDDP + Gemcitabine	30 (66)
FOLFOX/XELOX	6 (14)
GEMOX	4 (8)
Gemcitabine monotherapy	2 (4)
Other	4 (8)
1st line treatment discontinuation due to toxicity	
Yes	17 (37)
No	29 (63)
2nd line chemotherapy	
Irinotecan-based	22 (48)
Platinum-based	24 (52)

**Table 2:** Summary of laboratory tests’ distribution and deviations.

Laboratory Test	Mean Value	Median	Range
Hemoglobin (g/dL)	11.65	11.45	8.7–15.7
Neutrophil count (/mmc)	5630	4495	1820–21,270
Lymphocyte count (/mmc)	1323	1210	470–2880
Monocyte count (/mmc)	626	525	260–1240
Platelets count (/mmc)	213,000	194,500	96,000–571,000
Serum creatinine (mg/dL)	0.83	0.83	0.41–1.36
Total bilirubin (mg/dL)	0.53	0.45	0.2–1.5
GOT/GPT (U/L)	32/38	26/33	7–86/10–105
LDH (U/L)	260	219	96–701
Serum albumin (g/dL)	3.4	3.6	2.1–4.5
CEA (ng/mL)	16.31	3.05	0.3–246
Ca 19-9 (U/mL)	7762	206	2–100,000

**Table 3:** A Summary of the results of the ROC curve analysis.

Laboratory Test	Best Cut-Off (by Youden J-Index)	Sensitivity (%)	Specificity (%)	AUC (*p*)
Hemoglobin (g/dL)	>10 g/dL	100.0	50.0	0.763 (0.003)
Neutrophil count (/mmc)	≤5140/mmc	80.0	75.0	0.792 (0.001)
Lymphocyte count (/mmc)	>1810/mmc	24.0	100.0	0.580 (0.424)
Monocyte count (/mmc)	≤510/mmc	72.0	91.7	0.835 (<0.001)
Platelets count (/mmc)	>262,000/mmc	28.0	91.7	0.525 (0.804)
Serum creatinine (mg/dL)	≤0.9 mg/dL	76.0	66.7	0.667 (0.132)
Total bilirubin (mg/dL)	≤0.3 mg/dL	32.0	91.7	0.625 (0.179)
LDH (U/L)	≤417 U/L	96.0	33.3	0.547 (0.690)
Serum albumin (g/dL)	>3.5 g/dL	72.0	83.3	0.785 (0.002)
CEA (ng/mL)	≤3.3 ng/mL	83.3	54.5	0.697 (0.061)
Ca 19-9 (U/mL)	≤688 U/mL	75.0	72.7	0.723 (0.013)

**Table 4:** Results of univariate and multivariate analysis.

	Univariate Analysis			Multivariate Analysis	
Laboratory Test	Median OS (Months)	HR (95% CI)	*p*-Value	Exp (b) (95% CI)	*p*-Value
Hemoglobin > 10 g/dL					
Yes	12.31	0.014 (0.002–0.09)	<0.0001	0.1244 (0.0327–0.4737)	0.0023
No	2.26				
Neutrophil count ≤ 5140/mmc					
Yes	12.62	0.23 (0.09–0.58)	<0.0001	0.3035 (0.1352–0.6815)	0.0039
No	5.67				
Monocyte count ≤ 510/mmc					
Yes	12.62	0.36 (0.15–0.84)	0.0189	NA ^1^	NA ^1^
No	5.97				
Serum albumin > 3.5 g/dL					
Yes	9.24	0.38 (0.16–0.91)	0.0302	NA ^1^	NA ^1^
No	5.97				
Ca 19-9 ≤ 688 U/mL					
Yes	12.62	0.27 (0.10–0.68)	0.0057	NA ^1^	NA ^1^
No	5.97				

^1^ Values not included in the multivariate analysis because did not meet the statistical significance by Holm stepdown procedure.

## Data Availability

Data used as for the analyses that have been conducted are freely available upon reasonable request directed to the corresponding author.
